# Ferroelectricity-free lead halide perovskites†

**DOI:** 10.1039/c9ee00884e

**Published:** 2019-06-21

**Authors:** Andrés Gómez, Qiong Wang, Alejandro R. Goñi, Mariano Campoy-Quiles, Antonio Abate

**Affiliations:** Instituto de Ciencia de Materiales de Barcelona (ICMAB-CSIC), Campus UAB 08193 Bellaterra Spain agomez@icmab.es andres.gomez.rodríguez@csic.es; Helmholtz-Zentrum Berlin für Materialien und Energie Kekuléstrasse 5 12489 Berlin Germany qiong.wang@helmholtz-berlin.de antonio.abate@helmholtz-berlin.de; ICREA Passeig Lluís Companys 23 08010 Barcelona Spain; Institute of Advanced Energy Materials, Fuzhou University Fuzhou Fujian 350002 China; Department of Chemical, Materials and Production Engineering, University of Naples Federico II Piazzale Tecchio 80, Fuorigrotta Naples 80125 Italy antonio.abate@unina.it

## Abstract

Direct piezoelectric force microscopy (DPFM) is employed to examine whether or not lead halide perovskites exhibit ferroelectricity. Compared to conventional piezoelectric force microscopy, DPFM is a novel technique capable of measuring piezoelectricity directly. This fact is fundamental to be able to examine the existence of ferroelectricity in lead halide perovskites, an issue that has been under debate for several years. DPFM is used to detect the current signals, *i.e.* changes in the charge distribution under the influence of the scan direction and applied force of the atomic force microscope (AFM) tip in contact mode. For comparison, (i) we use DPFM on lead halide perovskites and well-known ferroelectric materials (*i.e.* periodically poled lithium niobate and lead zirconate titanate); and (ii) we conduct parallel experiments on MAPbI_3_ films of different grain sizes, film thicknesses, substrates, and textures using DPFM as well as piezoelectric force microscopy (PFM) and electrostatic force microscopy (EFM). In contrast to previous work that claimed there were ferroelectric domains in MAPbI_3_ perovskite films, our work shows that the studied perovskite films Cs_0.05_(FA_0.83_MA_0.17_)_0.95_Pb(I_0.83_Br_0.17_)_3_ and MAPbI_3_ are ferroelectricity-free. The observed current profiles of lead halide perovskites possibly originate from ion migration that happens under an applied electrical bias and in strained samples under mechanical stress. This work provides a deeper understanding of the fundamental physical properties of the organic–inorganic lead halide perovskites and solves a longstanding dispute about their non-ferroelectric character: an issue of high relevance for optoelectronic and photovoltaic applications.

## Abstract 2

We employ direct piezoelectric force microscopy to examine the ferroelectric response of halide perovskites and prove that halide perovskites are ferroelectricity free.

## Graphical abstract

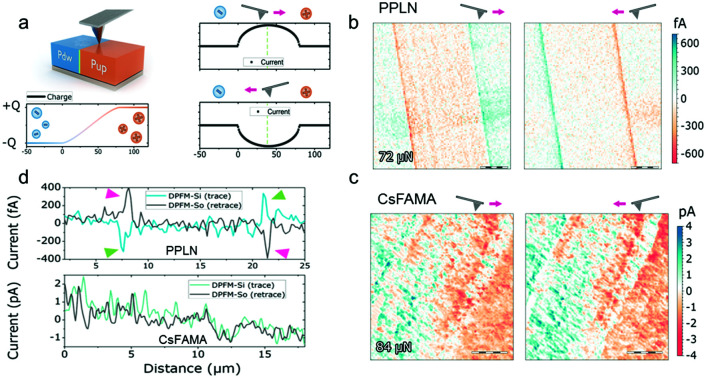

**Broader context**

Ferroelectric materials are characterised by a switchable macroscopic polarisation. A wide number of perovskite oxides have ferroelectric behaviour. Whether or not also lead halide perovskites are ferroelectrics has been under discussion since their successful application in photovoltaics and more recently in light emitting diodes. Though perovskite solar cells with a power conversion efficiency approaching their theoretical limit have been reported, the potential role of ferroelectricity in the photovoltaic behaviour is still puzzling. The high electronic and ionic conductivity in lead halide perovskites make the examination of ferroelectricity extremely complicated using conventional techniques, such as piezoresponse force microscopy. Experimental results from conventional methods often lead to misinterpretation. In this work, we employ direct piezoelectric force microscopy (DPFM) to examine the ferroelectric response of halide perovskites most commonly used in photovoltaics. In contrast with conventional techniques, DPFM collects a direct current signal response as a function of scan direction and applied force. By comparing to well-known ferroelectric materials, in our work we prove that halide perovskites are ferroelectricity free.

## Introduction

The successful demonstration of methylammonium lead iodide perovskite (MAPbI_3_) in photovoltaic devices^1–3^ has prompted strong interest in the fundamental properties of organic–inorganic lead halide materials.^4–6^ One of the currently open debates concerning the physical properties of lead halide perovskites focusses on the existence of a ferroelectric response. This is, indeed, a very relevant question for devices. It has been reported, for instance, that ferroelectric polarisation may favour charge generation and transport *via* adjustment of the band alignment, which leads to enhanced photovoltaic performance.^7–10^ On the other hand, it was suggested that a ferroelectric response might cause chemical segregation in perovskite films and hysteresis in the current–voltage (*JV*) scans of the corresponding solar cells.^11–14^

Before jumping to any conclusion, we need to look at the definition of the piezoelectric and ferroelectric effects and contrast them with other electrical properties. By definition, the piezoelectric effect corresponds to the generation of an electric charge in response to an applied mechanical stress.^15,16^ Due to the non-centrosymmetric structure, a dipole moment is induced in a piezoelectric material upon an applied mechanical stress, whereas ferroelectricity refers to the appearance of a spontaneous permanent polarisation which can be modulated by the application of an external electric field.^16^ Hence, a ferroelectric material can be simplified as being a piezoelectric material in which the polarisation can be reversed and, consequently, all ferroelectric materials are piezoelectric, but *vice versa* does not hold.^17–19^

Due to the potential importance of the existence of a ferroelectric response in MAPbI_3_, there is already a large, albeit contradicting, body of work on this topic.^20,21^ Though the most straightforward way to prove ferroelectricity is by showing switchable polarisation (*P*) under an applied electric field (*E*), which results in a commonly presented *P*–*E* hysteresis loop, these measurements are very challenging in lead halide perovskites, due to the relatively high electronic and ionic conductivities. Rakita *et al.*^13^ used the lossy part of the dielectric response and observed evident ferroelectric *P*–*E* behaviour from tetragonal MAPbI_3_ single crystals. Similar behaviour has been observed by Huang's group on MAPbI_3_ single crystals.^22,23^ However, no piezoelectric response was observed for polycrystalline films. Alvar *et al.*^24^ used dielectric constant measurements (*P*–*E* loops) for an Au/MAPbI_3_/Au device structure and concluded that MAPbI_3_ polycrystalline films are not ferroelectric. These studies would suggest that the weak ferroelectricity that was found in single crystals is not retained in polycrystalline films.

However, other work that adopted piezoresponse force microscopy (PFM), a standard method used to quantify ferroelectricity, reported a ferroelectric response in polycrystalline MAPbI_3_ films. For example, Kutes *et al.*^25^ studied the ferroelectric response using PFM and found the presence of ferroelectric domains in MAPbI_3_ films that are about the size of the grains (∼100 nm). The ferroelectric nature of the domains was also reported by Röhm *et al.*^11^ using PFM measurements. In our early work,^7^ based on PFM characterisation, we reported a value of *d*_33_ of around 5 pC N^−1^ for MAPbI_3_ films, which we found to be increased up to 25 pC N^−1^ under light illumination. The work of Liu *et al.*^14^ recently showed that the mere observation of a domain structure in PFM measurements is likely due to the peculiar interplay of several phenomena such as the ferroelastic nature of hybrid halide perovskites and chemical segregation effects. Moreover, the report of a sizeable electrostrictive response in MAPbI_3_ single crystals might provide an alternative explanation for electromechanical signals which are sometimes taken as evidence of ferroelectricity in AFM experiments.^23^

These contradicting reports lead us to ask ourselves if ferroelectricity is present in polycrystalline films, and if not, what is the origin of the PFM signal reported by several different groups. During our previous PFM ferroelectric study of MAPbI_3_ polycrystalline films,^7^ we noted that this classical measurement could lead to artefacts that were usually mistaken as ferroelectric signals in non-ferroelectric materials.^26–29^ Potential artefacts include electrostatic forces, ion migration, topography crosstalk, different conduction states, different chemical composition or temperature differences.^26,30^ Such effects can create a ferroelectric-like response in films that are not ferroelectric. To overcome the drawbacks of the PFM method, we have employed a novel methodology, direct piezoelectric force microscopy (DPFM),^31^ in which the piezoelectric effect is directly measured using an atomic force microscope (AFM). This technique measures the linearly dependent generated current due to a mechanical force applied, which makes it independent from the artefacts above as there are no other physical phenomena that can convert mechanical energy into electrical energy with linear dependency.

In this study, we show that for well-known ferroelectric materials, the DPFM signal: (i) shows a change in sign upon reversing the scan direction; and (ii) is proportional to the force as expected for a piezoelectric material. Similar effects are *not* observed when the DPFM technique is applied to films of the archetypal hybrid perovskite MAPbI_3_ and triple cation perovskite Cs_0.05_(FA_0.83_MA_0.17_)_0.95_Pb(I_0.83_Br_0.17_)_3_) (noted as the CsFAMA perovskite, where MA and FA stand for methylammonium and formamidinium, respectively). This strongly indicates that the perovskites are ferroelectricity free. Furthermore, we propose that the DPFM signals measured on the halide perovskites originate from ion migration induced by either a voltage or mechanical force applied by the AFM tip. Parallel experiments using other techniques such as PFM and electrostatic force microscopy (EFM) on MAPbI_3_ films of different grain sizes, film thicknesses, substrates and precursors are conducted and compared to the DPFM data. This set of experiments show the advantage of DPFM measurements over other techniques and help us to explain the “observed” ferroelectric domain reported in the literature.

## Results and discussion

**Fig. 1:**
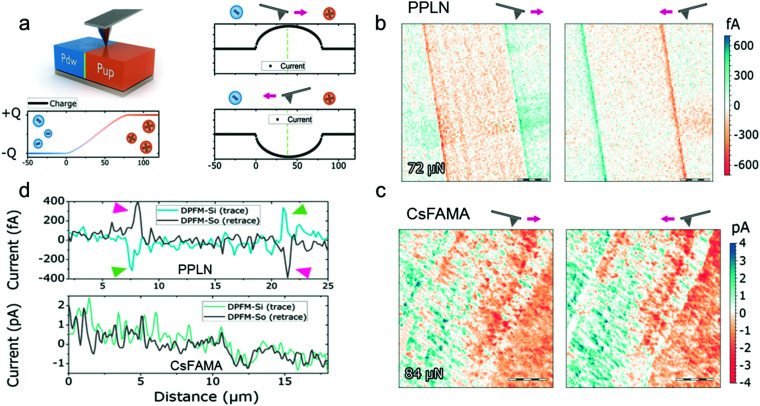
Scan direction dependence of the DPFM signals. (a) Scheme of the DPFM measurement of a ferroelectric sample (top left panel), with an antiparallel domain configuration, in which “Pdw” stands for “polarisation down” and “Pup” for “polarisation up”. Upon application of a suitable mechanical load, a negative charge is built up by the piezoelectric effect on the left side while a positive charge is built up at the right side (bottom left panel). The signal recorded, that is the current, is the derivative of the charge and it reverses its sign when the tip crosses different domains, depending upon the scan direction: the tip going from left to right (top right panel) and from right to left (bottom right panel). (b) DPFM images obtained for periodically poled lithium niobate (PPLN) with an antiparallel domain configuration. Scale bar: 5 μm. For PPLN, the current sign is reversed as the scan direction changes, exactly as expected for a ferroelectric domain structure. (c) DPFM images of the CsFAMA perovskite scanned under similar conditions to those of PPLN. Scale bar: 5 μm. The CsFAMA perovskite does not show a sign reversal of the DPFM signal, but rather the images for the two scan directions (left and right panels) are quite similar, which resemble typical current-sensing AFM mapping. (d) Random profiles extracted from PPLN (top panel) and the CsFAMA perovskite (bottom panel).

In our first experiment, we studied the DPFM signals under the influence of the scan direction. Fig. 1a depicts the working principle of DPFM, where a metallic tip is used to scan a set of different ferroelectric domain structures; the blue colour identifies a down domain polarisation (noted as “Pdw”), while the orange colour identifies an upwards polarisation (noted as “Pup”). The DPFM measurement was conducted with the AFM working in constant-force contact mode. As the force is constant, the number of piezo-generated charges is constant across the same ferroelectric domain, and it only changes when crossing the boundary between differently oriented domains. For instance, when the tip crosses the domain structures of Fig. 1a from left to right, a current is generated as the tip passes from the region of negative charge accumulation (left side) to that of positive charge accumulation (right side). When the tip scans back, the current changes its sign and flows in the opposite direction, as the tip goes from a positive charge accumulation (right side) to a negative charge accumulation region (left side). Such an imaging mechanism is unique compared with any other techniques available. Moreover, the only physical phenomenon that can produce such images is ferroelectricity because if a physical phenomenon could induce a contrast reversal, such physical phenomena have to be linearly dependent on the applied force and independent of the number of scans, which indicates such a possible artefact has to convert, in an unlimited manner, mechanical energy into electrical energy. The imaging mechanism of DPFM can be experimentally visualised for any ferroelectric material. Fig. 1b shows the two images of periodically poled lithium niobate (PPLN) when the tip scans from left to right (left panel) and backwards (right panel). It presents clear current peaks in the areas where the tip crosses antiparallel ferroelectric domains. More importantly, the current sign is changed for the two scanning directions: left to right, named DPFM-Signal Input (DPFM-Si, left panel), and right to left, named DPFM-Signal Output (DPFM-So, right panel).^31^ The DPFM-Si and DPFM-So images are approximately mirror-reflections of each other. We then performed a similar experiment on the CsFAMA perovskite. The results are given in Fig. 1c. Significantly different from the DPFM images of PPLN given in Fig. 1b, the current in the CsFAMA perovskite does not show clear changes as the scan direction reverses. Essentially, the DPFM-Si (left panel) and DPFM-So (right panel) images in Fig. 1c look very similar to each other. This means that the AFM probe reads a current flowing through the CsFAMA perovskite, but there is no inversion of the current upon scan direction changes, a response that does not indicate a ferroelectric material.^11^ Discussion on the origin of the current response in the CsFAMA perovskite will be given in the following paragraphs. Fig. 1d displays representative profiles extracted from the DPFM images measured for PPLN (top panel) and the CsFAMA perovskite (bottom panel). It clearly shows that the current in PPLN is inverted when the scanning direction switches, while the current trace in the CsFAMA perovskite remains the same in both scan directions.

In our second experiment, we studied the DPFM signals under the influence of the applied force. This experiment relies on the fact that a ferroelectric material is also piezoelectric.^32^ For a piezoelectric material, the recorded current should increase linearly with the applied load. Therefore, in the next series of experiments, we investigate the response of our samples as a function of the applied force. We first conducted the experiment on reference materials, namely PPLN (*d*_33_ = 14 pC N^−1^), commercially available lead zirconate titanate (PZT) 5A4 (*d*_33_ = 460 pC N^−1^) and PZT 503 (*d*_33_ = 500 pC N^−1^). *d*_33_ represents the piezoelectric constant of the materials, which accounts for electric dipole generation parallel to the poling direction when the force is also applied parallel to the poling direction.^33^

**Fig. 2:**
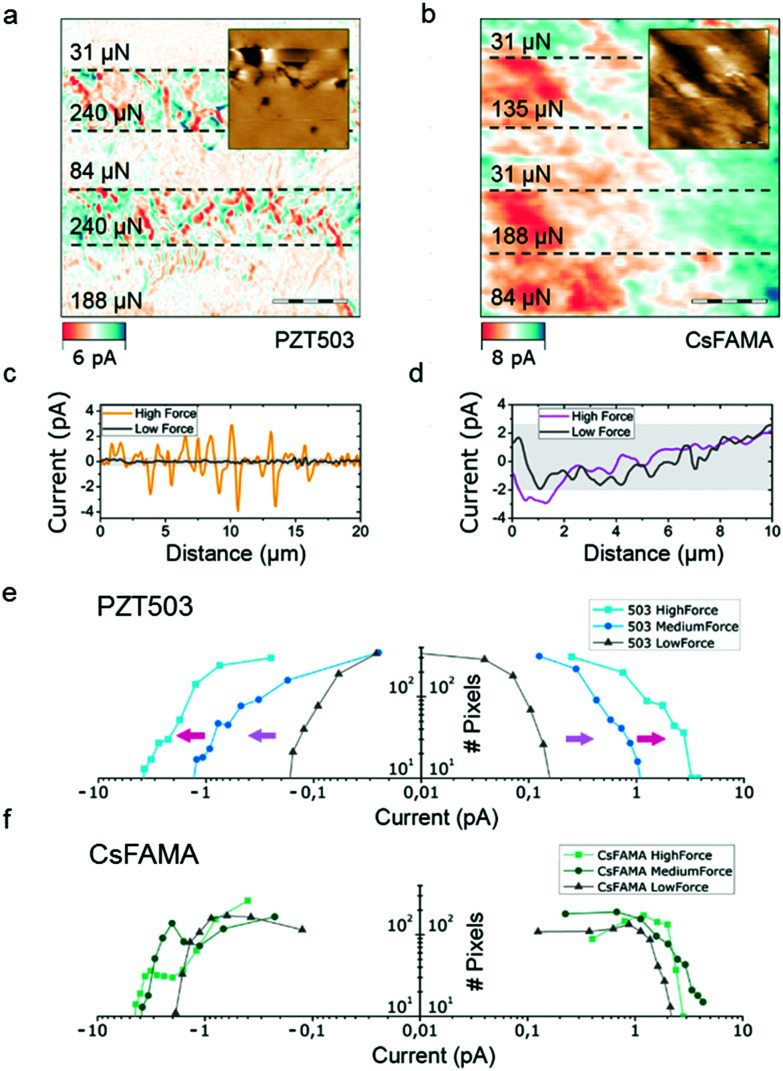
Force dependence of the DPFM signals: the (a) PZT 503 reference sample and (b) CsFAMA perovskite. Dashed lines represent changes in the applied force. Randomly selected profiles for the PZT 503 reference sample (c) and the CsFAMA perovskite (d) at a high and a low applied force. For the known ferroelectric materials, the input signal changes dramatically with the load exerted by the tip. However, for the CsFAMA perovskite, there is no appreciable dependence on the applied load. Histograms (e) and (f) extracted from (a) and (b), comparing the PZT 503 reference sample and the CsFAMA perovskite. The current recorded (*x*-axis) for PZT 503 shows a clear dependence on the applied load, while the current in the CsFAMA perovskite does not show any correlation.

Fig. 2 corresponds to the DPFM-Si scan frame obtained in trace mode. The insets correspond to the topography frame acquired simultaneously. While performing the trace scan, the force exerted on the material was sequentially changed, as marked by the black dashed lines in Fig. 2a and b. The scan starts from the bottom to the top. In the case of PZT, a thermal treatment was employed to heat the sample above the Curie temperature, revealing the natural domain structure for this material.^31,34^Fig. 2a shows that for the known ferroelectric samples, *i.e.* PZT 503, the recorded current is highly dependent upon the applied load: the higher the load, the higher the current. The topography frame of PZT 503 in the inset to Fig. 2a shows that the ferroelectric domain configuration in PZT 503 consists of antiparallel domains that were previously periodically poled. (To minimise any instrumentation related artefact, two different tips are used for this measurement. See the Methods section and Fig. S1, ESI.†) The dependence of the current signals on the applied load for PZT 503 becomes more prominent when extracting random profiles obtained at the highest and lowest loads (Fig. 2c). Considering PZT 503 has a relatively large piezoelectric constant value (a *d*_33_ of around 500 pC N^−1^), reference samples of small piezoelectric constant value are also studied. PPLN (*d*_33_ = 14 pC N^−1^) is also measured in this experiment. As shown in Fig. S2 (ESI†), a clear difference in the recorded current can be observed for PPLN, though the current change is more pronounced in PZT 503. Herein, we have confirmed the dependence of the recorded current on the applied load for piezoelectric materials in DPFM measurements. However, such dependence is not observed for the CsFAMA perovskite, as illustrated by the DPFM images of the CsFAMA perovskite, shown in Fig. 2d.

To further examine this dependence between current and applied force, we obtained pixel histograms to compare the CsFAMA perovskite and PZT 503. The histograms in units of current are obtained by analysing the pixel value of each image, and then plotting the number of pixels for a given range, as illustrated in Fig. 2e. The histograms of the PZT 503 sample show that there is a dramatic change in current depending on the applied load. A calculated linear dependency is shown in Fig. S3 (ESI†). In the case of PPLN and PZT, the signal increases linearly with the applied force, whereas the CsFAMA perovskite (Fig. 2f) does not show such a dependency. Despite the lack of dependency of the current on the applied bias in the CsFAMA perovskite, one may argue that the dark current could mask the piezoelectric generated current. To investigate if this was the case, we calculated the derivative of the current profiles, as the piezoelectric generated current is read as “peaks” in contrast to the dark current in the whole scanned area. Fig. S3 (ESI†) shows that the derivative of the current profiles is rather flat compared to the significant vibration observed in PZT. More importantly, we also calculated the theoretical piezo-generated current peaks with the assumption that the CsFAMA perovskite had a *d*_33_ constant of 5 pC N^−1^, and superimposed it onto the recorded signal. In both favourable and unfavourable cases, which depends upon the tip–sample contact area, the piezoelectric signal should appear in our data, which is not what our data reveal. Another essential aspect relies on the ferroelectric domain orientation; in-plane or out-of-plane should give a similar peak current distribution where the main differences would be the quantification of the *d*_31_ constant rather than the *d*_33_ constant, despite head-to-head to head-to-tail domain configurations. As a result, our experiment shows that the CsFAMA perovskite does not show a detectable piezoelectric effect.

So far we have shown that the behaviour of CsFAMA perovskite films in DPFM experiments differs significantly from that of conventional ferroelectric materials, *i.e.* PPLN and PZT studied in this work. This fact powerfully speaks against the claims of ferroelectricity in the lead halide perovskites. Thus we are led to the conclusion that the observed DPFM response of the halide perovskites has a different origin. In the remaining, we will set the grounds of an interpretation of the DPFM results in terms of ion migration effects, triggered either by mechanical stress induced by force exerted by the AFM tip or by electric fields associated with an applied tip bias. Our speculation can, moreover, help to understand the origin of the signal detected in conventional PFM experiments.

**Fig. 3:**
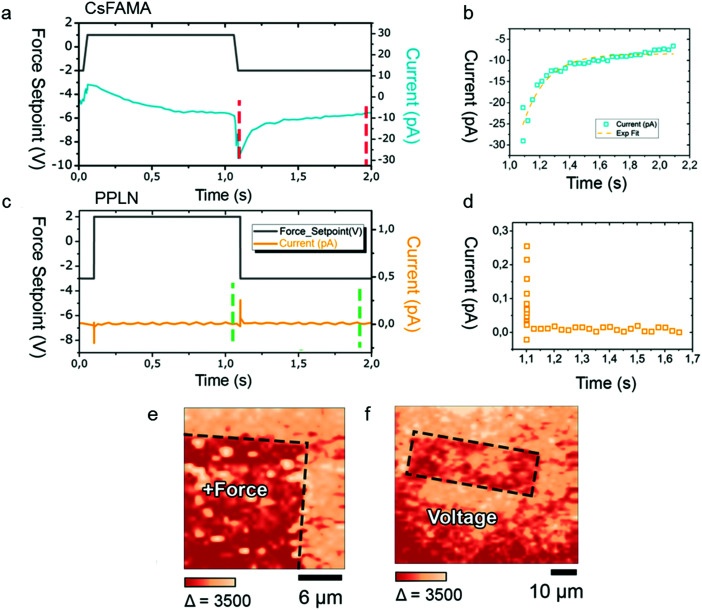
DPFM measurement in static mode and the corresponding spectroscopic study. (a) Force *versus* distance curve performed at a single spot of the CsFAMA perovskite with simultaneous recording of current signals. The adjustment in the force, as indicated by the solid black line, will cause a current signal corresponding to the piezoelectric effect. The value of the current signal will reset to the zero level rapidly once the force is applied steadily again. The curve plotted here is the average of 16 different traces obtained at different spots on the CsFAMA perovskite to average sample inhomogeneity. (b) Zoom-in of the time region between the dashed lines, where the CsFAMA perovskite exhibits an extremely slow decay of the current signal on the time scale of ∼1 s. (c) Force *versus* distance curve performed at a single spot of the lithium niobate sample (PPLN) with simultaneous recording of current signals. (d) Zoom-in of the time region between the dashed lines, where the piezoelectric material lithium niobate shows a much faster decay of current on a time scale below the limit set by our transimpedance amplifier of ∼6 ms. (e and f) Photoluminescence (PL) mapping of the CsFAMA perovskite sample of different regions which were previously pre-patterned either by application of a force (e) or a bias (f), using the AFM tip.

In the next experiment, we performed an *in situ* investigation of the change in current when varying the applied load of the AFM tip. We want to emphasise that piezoelectric materials produce current only when the force is changed, but not if the material is held at a fixed force. We monitored the current changes in the DPFM experiment, where the applied load was varied while the AFM tip is kept static at the surface of the sample. For this kind of experiments, DPFM measurements were conducted on an array of 4 × 4 points, 16 curves in total, in an area of 10 × 10 μm. For each curve, the AFM tip was held at one spot while the force applied to the tip was changed as indicated by the black line in Fig. 3a and c. In this force profile, the force starts at a constant value of 20 μN and then increases to 226 μN in a 50 ms time interval. After holding the tip at 226 μN for 1 s to stabilise the system, the force is decreased to its initial value of 20 μN in 50 ms and stays there for 1 s. During the whole cycle, the current changes at the tip are monitored, as illustrated by the blue line in Fig. 3a and the orange line in Fig. 3c. These traces are the result of averaging 16 curves obtained at the 4 × 4 points in each case. The individual 16 curves are provided in Fig. S4 (ESI†). For piezoelectric materials, *i.e.* PPLN in this study, the current signal holds at zero for the period when the force is kept constant and has one negative peak and one positive peak as the force is increased from 20 to 226 μN and decreased from 226 to 20 μN, respectively (Fig. 3c). Notably, the current changes from its peak value to zero in milliseconds (ms) (Fig. 3d). In this case, the response is limited by the time constant of our amplifier of 6 ms. However, DPFM measurements on the CsFAMA perovskite (Fig. 3a) yield a completely different picture compared to PPLN (Fig. 3c): the current increases from a negative value and reaches a plateau when the force is increased from 20 to 226 μN, which is followed by a gradual decrease in current when the force is kept constant at 226 μN, and then the current drops to its lowest value and slowly increases to a constant value below zero, as the force is dropped to and held at 20 μN. For clarity, the current drop process is zoomed in, as illustrated in Fig. 3b. An exponential growth function was fitted to the data points yielding a recovery time of ∼150 ms. Such a slow recovery of the current is not compatible with a piezoelectric effect arising from lattice distortions.

To further investigate the origin of the peculiar current profile of the CsFAMA perovskite (Fig. 3a), we performed experiments combining the DPFM technique with micro-photoluminescence (PL) measurements. In this case, a square area of 40 × 40 μm^2^ was scanned in DPFM under a constant force, followed by PL mapping of the same area. The PL spectra of the pre-patterned sample are given in Fig. S5 (ESI†). Fig. 3e shows that after the application of a constant force of 226 μN during the DPFM measurement, a lower PL intensity is observed inside the square area compared to outside the imprinted area of the CsFAMA perovskite. To better understand this difference, we scanned a rectangular area of the same sample, applying a suitable electrical bias to the AFM tip, while the force is set to the minimum value needed to ensure a good tip–sample mechanical contact (Fig. 3f). It shows that the rectangular area has a lower PL intensity than its adjacent area where no force is applied. The similarity in the results of PL mapping for the CsFAMA perovskite after the application of a force or bias is undoubtedly an indication of conventional underlying physical processes.

In the pre-patterned regions, irrespective of its origin by applying a voltage or exerting a constant force with the AFM tip, the reduction of the PL intensity means less efficient radiative recombination. There are several mechanisms acting to the detriment of radiative recombination, which could be simultaneously at work in the pre-scanned regions.^35^ The PL intensity might be reduced by the formation of defects, mainly ionic vacancies, near the sample surface, resulting from the movement of ions in the perovskite induced by either the applied voltage or bias. On the other hand, the moving ions would influence Fermi-level pinning, creating a space-charge layer close to the surface, within which photo-generated electron–hole pairs are dissociated by the built-in electric field. In this way, radiative recombination becomes largely impaired in the space charge region, leading to overall less intense PL emission. Similar effects have also been observed for crystalline Si wafers with different kinds of surface passivation.^36^ Interestingly, the PL intensity is lower in the region where the applied voltage changed sign such that ions are no longer attracted but repelled from the surface. This behaviour agrees with what is expected for both a higher defect concentration and a stronger built-in electric field close to the surface.

We want to emphasise that Luo *et al.*^37^ also reported PL changes of MAPbBr_3_ single crystals before and after an applied bias. They found that when a negative bias of −2 V was applied to a MAPbBr_3_ single crystal, a decrease in PL intensity was observed in the applied area, whereas when a positive bias of 2 V was applied, an increase in PL intensity was observed. Their observations upon application of a bias agree well with our findings for CsFAMA films. The same mechanisms based on the movement of halide atoms are proposed in Luo's work^37^ to account for the changes in PL intensity in the biased areas. Similarly, it is reasonable to believe that the reduced PL intensity under the applied force in our DPFM measurements is caused by the ion movement of iodide away from the surface. Finally, we point out that a bias voltage has been recently used to switch on and off the photoluminescence from a MAPbI_3_ film,^40^ which enabled monitoring of ion migration and the accompanying ionic vacancy drift, accounting for the time dependence of the electrical current in the device. It is more representative of sluggish charge motion which can be ascribed to ion migration. Meanwhile, several studies have shown that ion movement inside lead halide perovskites is taking place when an external bias is applied.^35,38–40^ Therefore, we propose that the current profile in Fig. 3a for the CsFAMA perovskite is likely coming from ion migration mediated by strain.^41,42^ As a result, we observe: (i) a negative current when a force of 20 μN was initially applied to the sample, triggering ion movement; (ii) an increase in current close to zero as the force was gradually increased from 20 μN to 226 μN in 50 ms, since more ions were activated during this process; (iii) a decrease in current when the force was kept constant at 226 μN, due to a decrease in concentration of available local ions; (iv) a drop in current as the force decreased from 226 μN to 20 μN, leading to less driving force for ion movement and (v) a gradual increase to a plateau as the force was held at 20 μN (gradual movement of ions under a low force).

**Fig. 4:**
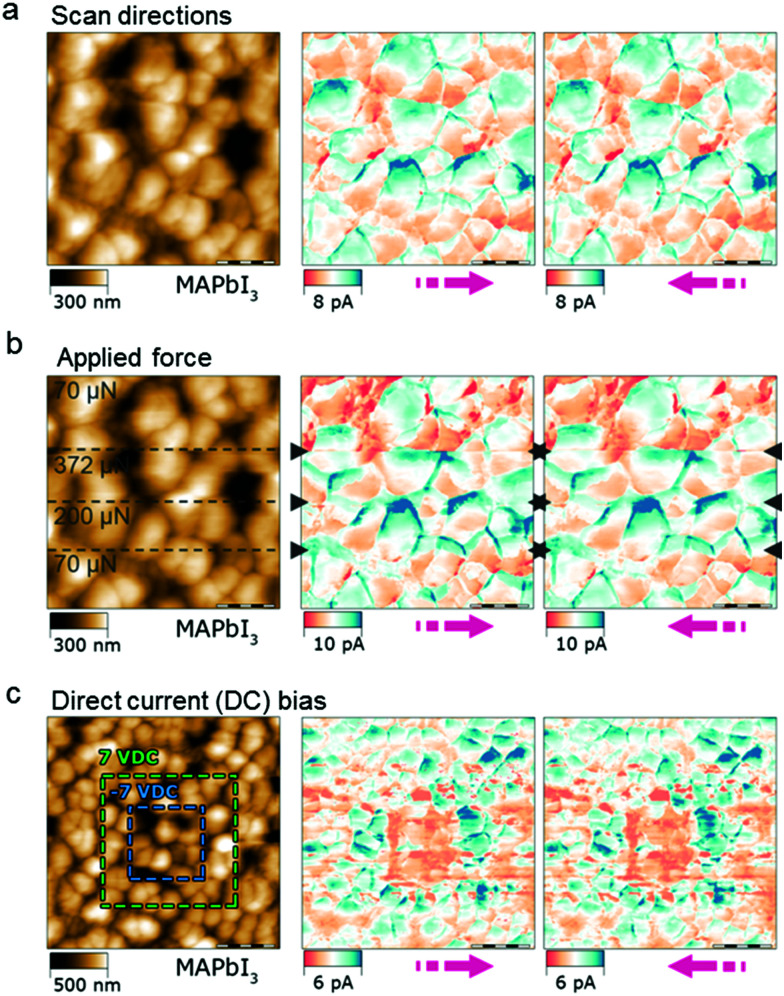
DPFM measurements of the MAPbI_3_ perovskite. (a) Dependence of the DPFM signal on the scan direction: topography (left), DPFM-Si (middle) and DPFM-So (right) of a MAPbI_3_ perovskite film. (b) Dependence of the DPFM signal on the applied force (changes in force are indicated with black dashed lines): topography (left), DPFM-Si (middle) and DPFM-So (right) of the MAPbI_3_ perovskite film. (c) DPFM images of the MAPbI3 perovskite film after the application of a direct current (DC) bias: topography (left), DPFM-Si (middle) and DPFM-So (right).

By far, our work has demonstrated that the CsFAMA perovskite is ferroelectricity free, which is in agreement with the fact that mixed cations and mixed halide perovskites exhibit spatial inhomogeneities of cations and halides in the film, as well as different polarities.^43^ However, this might not be the case in the MAPbI_3_ perovskite because of the influence of local polarity of MA^+^ cations, which may cause a polarisation orientation in the crystal structure.^44,45^ Thus the MAPbI_3_ perovskite may hold a ferroelectric property, but this conclusion is still very much under debate.^46–48^ In the next experiment, we expanded our study to MAPbI_3_ to see if our conclusion could be applied to the MAPbI_3_ perovskite. Fig. 4a shows that the MAPbI_3_ perovskite film does not exhibit current inversion with the scan direction. DPFM-So and DPFM-Si images essentially look the same, as is observed in the CsFAMA perovskite (Fig. 2b). The force dependence of this current is studied in the same region (Fig. 4b). The horizontal dashed lines in Fig. 4b (left panel) represent different forces applied to the material, while the DPFM-So and DPFM-Si images are depicted in the middle and right panels, respectively. It shows barely any change in the current recorded at three applied forces. Fig. S6 (ESI†) shows the current signal at 70 μN superimposed onto that at 372 μN, where the two current curves almost overlap with each other. These results are extremely similar to what we have observed for the CsFAMA perovskite. In addition, we performed another experiment, where we applied a positive bias of +7 V on the outside square area (green dashed line in Fig. 4c left panel) and then a negative bias of −7 V on an inside square area (blue dashed line in Fig. 4c left panel), and then we performed the DPFM measurement for two scan directions. As shown in Fig. 4a, DPFM-So and DPFM-Si have very similar current distributions, not influenced by the scan direction. Moreover, as discussed above, for a ferroelectric material, a current profile following the inside square line will appear, while in other areas the current should be zero. However, the current profile of the MAPbI_3_ perovskite shows a high value for the inside square area and a low value for the area between the inside and outside square area. As we discussed for the CsFAMA perovskite, the current profile of MAPbI_3_ can be explained well by ion migration. For the outside and inside square areas, where a positive and negative bias was applied, respectively, the direction of ion migration is the opposite. Here we applied first the positive bias and then the negative bias. Therefore, the ions inside the negative bias area will move from the surface to the bulk of the MAPbI_3_ perovskite, whereas the ions outside the negative bias area but inside the positive bias area will move from the bulk to the surface of the MAPbI_3_ perovskite. As a result, the difference in the current distribution is determined by the area of the applied bias rather than the perimeter where the applied bias was changed.

**Fig. 5:**
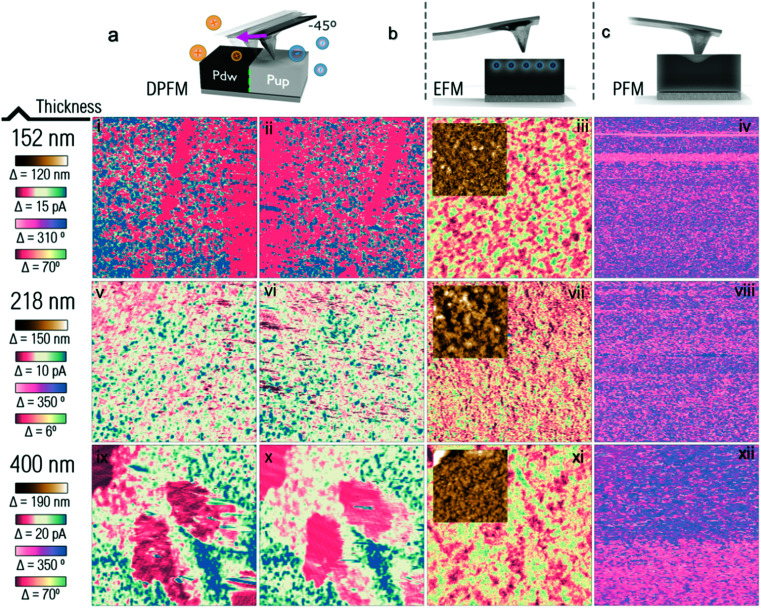
Scheme of the three AFM modes DPFM (a), EFM (b) and PFM (c) with the measurement results of the MAPbI_3_ perovskite at a film thickness of 152 nm ((i): scanning from left to right, and (ii): scanning from right to left for DPFM measurements; and (iii) and (iv) for EFM and PFM measurements, respectively), 218 nm ((v): scanning from left to right, and (vi): scanning from right to left for DPFM measurements; and (vii) and (viii) for EFM and PFM measurements, respectively), and 400 nm ((ix): scanning from left to right, and (x): scanning from right to left for DPFM measurements; and (xi) and (xii) for EFM and PFM measurements, respectively). Insets given in (iii), (vii), and (xi) are the topography channel of EFM images of the samples.

In the next paragraphs, we examined the ferroelectricity in MAPbI_3_ perovskites of different grain sizes, film thicknesses, texture features and substrates using two additional AFM modes: PFM and EFM in parallel to DPFM. We started with samples with film thicknesses ranging from 152 nm to 400 nm, adjusted by changing the spin-coating speed during the deposition of the MAPbI_3_ perovskite. The cross-sectional images and surface morphology of the perovskite films can be found in the ESI† (Fig. S9) with the grain size distribution measured using Image J software given in Fig. S10 (ESI†). The DPFM, EFM and PFM measurements of the samples are given in Fig. 5. The DPFM measurement of the MAPbI_3_ perovskite of a film thickness of 152 nm given in Fig. 5(i) and (ii) shows that no current inversion is observed when the scan direction is flipped, which indicates that the MAPbI_3_ perovskite of a film thickness of 152 nm does not show ferroelectricity. Similarly, Fig. 5(v), (vi), (ix) and (x) imply that MAPbI_3_ perovskites of a film thickness of 218 nm and 400 nm are ferroelectricity-free. The EFM and PFM images given in Fig. 5(vii), (viii), (xi) and (xii) do not show the ferroelectric domains that were reported in the literature.^11^

**Fig. 6:**
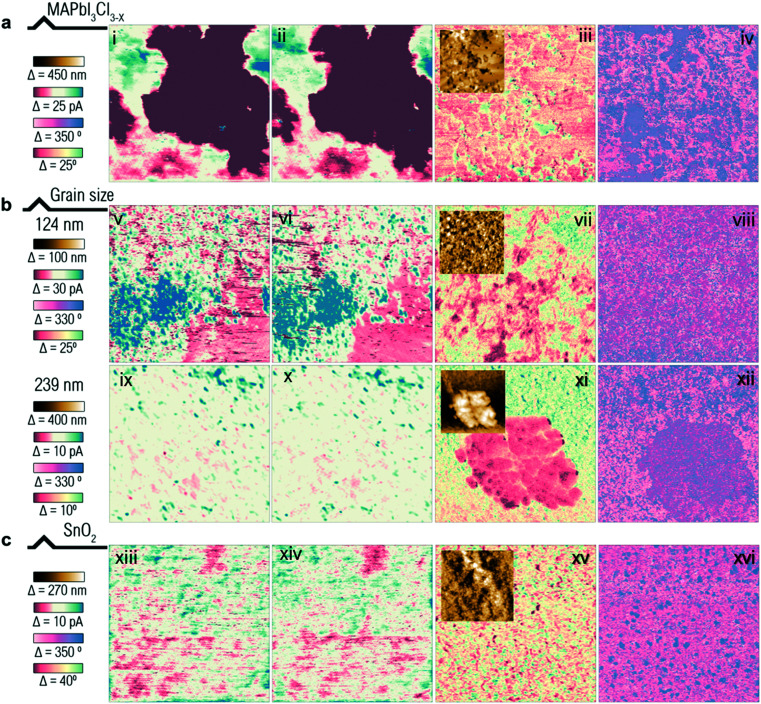
Matrix panels of AFM advanced modes DPFM, EFM and PFM of the MAPbI_3_ perovskite prepared from PbCl_2_ ((i): scanning from left to right, and (ii): scanning from right to left for DPFM measurements; and (iii) and (iv) for EFM and PFM measurements, respectively), at a grain size of 124 nm ((v): scanning from left to right, and (vi): scanning from right to left for DPFM measurements; and (vii) and (viii) for EFM and PFM measurements, respectively), and at a grain size of 239 nm ((ix): scanning from left to right, and (x): scanning from right to left for DPFM measurements; and (xi) and (xii) for EFM and PFM measurements, respectively), and the tin oxide covered conductive glass ((xiii): scanning from left to right, and (xiv): scanning from right to left for DPFM measurements; and (xv) and (xvi) for EFM and PFM measurements, respectively). Insets given gave in (iii), (vii), (xi) and (xv) are the topography channel of EFM images of the samples.

The MAPbI_3_ perovskite prepared from PbCl_2_ was examined with the three AFM modes to examine the influence of the texture of the perovskite film on the measurement. The scanning electron microscopy (SEM) images in Fig. S11 (ESI†) show that it has a much bigger grain size with a different texture feature from that of the MAPbI_3_ perovskite prepared from PbI_2_ using the anti-solvent method.^49^ The X-ray diffraction (XRD) pattern given in Fig. S15 (ESI†) shows that the MAPbI_3_Cl_3−*x*_ perovskite presents a pronounced grain orientation along the (110) facet. The DPFM measurements given in Fig. 6(i) and (ii) show that a much higher current is observed in the MAPbI_3_Cl_3−*x*_ perovskite. However, no inversion of current is found when the scan direction is switched from left (Fig. 6(i)) to right (Fig. 6(ii)). As a result, we conclude that the sample is ferroelectricity-free, similarly to the reference MAPbI_3_ perovskite given in Fig. 4. Interestingly, the EFM and PFM measurements of the MAPbI_3_Cl_3−*x*_ perovskite given in Fig. 6(iii) and (iv) show both amplitude and phase signals that correlate with possible domain patterning.^11^ This further supports our statement that DPFM shows advantages over PFM and EFM, the latter being highly influenced by artefacts and by ion movement in perovskite films.^13,26,50^

MAPbI_3_ perovskites of average grain sizes of 124 nm and 239 nm are studied in the three advanced AFM modes. The surface morphology and film thickness of the samples are given in Fig. S12 (ESI†) with the grain size distribution measured using Image J software given in Fig. S13 (ESI†). The DPFM measurements of the samples given in Fig. 6(v) and (vi) for the MAPbI_3_ perovskite of an average grain size of 124 nm and Fig. 6(ix) and (x) for 239 nm do not show current signal inversion when the scan direction is changed from left to right. As a result, MAPbI_3_ perovskites of small and large grain sizes do not show ferroelectricity. Interestingly, a current signal is revealed both in EFM and PFM images of the MAPbI_3_ perovskite of a large grain size of 239 nm, as shown in Fig. 6(xi) and (xii). With the topography channel, shown as an inset in Fig. 6(xi), we can spot that crosstalk could be present for the PFM and EFM frames, as they look similar to the topography channel.

To exclude the possible influence of the substrates, MAPbI_3_ perovskite deposited on SnO_2_ covered conductive glass is prepared and compared to the MAPbI_3_ perovskite that is deposited on PEDOT:PSS (poly(3,4-ethylenedioxythiophene) polystyrene sulfonate) covered conductive glass (detailed information on the samples’ preparation can be found in the Experimental section). Fig. 6(xiii) and (xiv) show that no current signal inversion is observed in the DPFM measurement, which indicates that the MAPbI_3_ perovskite deposited on an electron selective contact, similar to the MAPbI_3_ perovskite deposited on a hole selective contact, does not show ferroelectricity. Though no domain feature correlated with the ferroelectric property is observed in the EFM measurement (Fig. 6(xv)), a phase contrast can be clearly identified in the PFM measurement (Fig. 6(xvi)). This further shows that misleading messages can be obtained from conventional ferroelectric measurements.

Based on the above discussion, it is safe to conclude that MAPbI_3_ polycrystalline films are ferroelectricity free. However, it is worth noting that for MAPbI_3_ single crystals, the observation of ferroelectricity has been reported. The difference has its origin in the random distribution of grain orientations in polycrystalline films.^22^

## Conclusion

In summary, we have used a powerful novel technique, DPFM, to probe the possible appearance of ferroelectricity in two lead halide perovskites, namely CsFAMA and MAPbI_3_. We compared the DPFM measurement with conventional ferroelectric techniques, *i.e.* EFM and PFM, for MAPbI_3_ perovskites of different grain sizes, film thicknesses, textures and substrates. This work demonstrates that MAPbI_3_ perovskite films are ferroelectricity-free, which is not influenced by the grain size, film thickness, texture of the grains and electron or hole selective contacts beneath. Finally, this work helps to solve the debate of whether or not lead halide perovskites are ferroelectric. Moreover, it contributes to understanding the causes of current signals reported in previous ferroelectric studies of lead halide perovskites. This work also provides a new tool to examine ferroelectric behaviour in thin films and thus will inspire more discussion on the fundamental physical properties of lead halide perovskites, in particular, and their influence on photovoltaic performance, in general.

## Experimental methods

### Sample preparation

The CsFAMA triple cation perovskite was prepared adopting the method reported in our previous work.^49^ Briefly, 1.5 M lead iodide (PbI_2_) and lead bromide (PbBr_2_) stock solutions in a mixture solvent of dimethylformamide (DMF) and dimethyl sulfoxide (DMSO) at a volume ratio of 4 to 1 were prepared freshly. A stock solution of 1.5 M cesium iodide (CsI) in DMSO was prepared at the same time. Then 0.0773 g of methylammonium bromide (MABr) and 0.223 g of formamidinium iodide (FAI) were weighted to make 1.24 M FAPbI_3_ and MAPbBr_3_ solutions separately. PbI_2_ to FAI and PbBr_2_ to MABr were kept at a molar ratio of 1.09 to 1. Then FAPbI_3_ was mixed with MAPbBr_3_ at a volume ratio of 83 : 17, and then the solution was mixed with CsI at a volume ratio of 95 to 5. The obtained CsFAMA triple cation perovskite was spin-coated on tin oxide covered ITO at a speed of 1000 rpm for 5 s and 6000 rpm for 35 s. Chlorobenzene was used as the anti-solvent, dripped 5 s before the end of the spin-coating process. Then the perovskite was annealed on a hot plate at 100 °C for 1 hour. Tin oxide was prepared by spin-coating 17 mg ml^−1^ tin chloride dehydrate in isopropanol at 3000 rpm for 30 s, and then annealed at 180 °C for 1 hour.

The MAPbI_3_ perovskite was prepared by mixing PbI_2_ with methylammonium iodide (MAI) at a molar ratio of 1 to 1. In particular, 1.3 M PbI_2_ in DMSO was prepared as the stock solution. The precursor was spin coated on PEDOT:PSS (poly(3,4-ethylenedioxythiophene) polystyrene sulfonate) covered conductive glass (ITO, indium-doped tin oxide covered glass) at a speed of 1000 rpm for 5 s and then 4000 rpm for 20 s. Chlorobenzene was dripped 5 s before the end of the program. Then the sample was annealed on a hot plate at 100 °C for 60 min. PEDOT:PSS was spin-coated on pre-cleaned ITO at a speed of 4000 rpm for 40 s and then annealed on a hot plate at 160 °C for 20 min. MAPbI_3_ perovskites of different film thicknesses are deposited at different spin-coating speeds. MAPbI_3_ perovskites of different grain sizes are controlled by the annealing temperature. The surface morphology for the grain size and cross-sectional images for the film thickness of the MAPbI_3_ perovskite films can be found in the ESI.† Tin oxide covered ITO was prepared by dissolving 15 mg ml^−1^ tin chloride dehydrate in isopropanol and spin-coating at 3k rpm, followed by annealing at 180 °C for 1 h. The MAPbI_3_Cl_3−*x*_ perovskite was prepared by mixing MAI and PbCl_2_ in a molar ratio of 3 to 1 with a total mass weight of 40 wt% in DMF solvent, and then spin-coating at 2k rpm, followed by annealing at 100 °C for 45 min.

### AFM characterization

The measurements were performed using a Keysight 5500LS while a special ultra low leakage amplifier is used in transimpedance configuration (TIA) with part number ADA4530-1. The TIA amplifier is populated with a 10 GOhm feedback resistor, which give us a current noise level of 
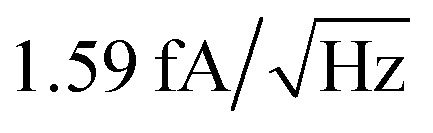
, while the leakage current in these conditions is maintained at less than 10 fA – data obtained from the amplifier datasheet of Analog Devices Inc. At the output of the current amplifier, a cascade voltage amplifier increases the overall gain of the system. The RC time constant of the amplifier is estimated at 6 ms, which equals a strain feedback capacitor of 0.1 pC. The two cascade amplifiers are calibrated with a known test resistor and a known bias applied, see ESI† Fig. S7 to see the calibration curves. In order to calculate the force used, we employed a standard method based on force *vs.* distance curves, see ESI† Fig. S8, performed in spectroscopy mode to acquire the tip deflection sensitivity. This value is used to convert the difference between the free deflection value and the set point value into force units, by using the cantilever spring constant. All the measurements are performed in low humidity conditions, less than 8%, to avoid possible electrochemical effects that are not explored in this text. The rest of the conditions for each frame of the text are given below:

Fig. 1b: 256 × 256 px, 20 × 20 μm, 34.75 μm s^−1^, RMN-25pt200-h

Fig. 1c: 128 × 128 px, 30 × 30 μm, 197.8 μm s^−1^, DDESP-V2

Fig. 2a: 128 × 128 px, 30 × 30 μm, 197.8 μm s^−1^, DDESP-V2

Fig. S1: 256 × 256 px, 30 × 30 μm, 64.98 μm s^−1^, RMN-25pt200-h, ESI†

Fig. 2b: 256 × 256 px, 30 × 30 μm, 64.98 μm s^−1^, RMN-25pt200-h

Fig. 2c: 256 × 256 px, 30 × 30 μm, 64.98 μm s^−1^, RMN-25pt200-h

Fig. 2d: 256 × 256 px, 10 × 10 μm, 15.49 μm s^−1^, RMN-25pt200-h

Fig. 4a: 256 × 256 px, 20 × 20 μm, 45.25 μm s^−1^, RMN-25pt200-h

Fig. 4b: 256 × 256 px, 20 × 20 μm, 45.25 μm s^−1^, RMN-25pt200-h

Fig. 4c: 512 × 512 px, 40 × 40 μm, 79.61 μm s^−1^, RMN-25pt200-h

Fig. 5 and 6: 1. DPFM images: 256 × 256, 128.3 μm s^−1^. RMN-25ptir200-h 2. EFM images, 15 × 15 μm, are obtained in single-pass EFM mode. The cantilever is excited at a constant frequency of 20 kHz while an AC bias amplitude of 4–5 V is applied directly to the tip (Nanosensors EFM, a PtIr coated tip with a cantilever spring constant of 3 N m^−1^). PFM images are acquired using a Nanosensors EFM, exciting the lever with 20 kHz and a VAC of 3–5 V.

### Optical characterization

Photoluminescence (PL) was measured with a WITec alpha 300 RA+ confocal setup using either the 633 nm line of a He–Ne laser or the 785 nm line of a diode laser for excitation. We employed an Olympus objective with 100× magnification and a NA = 0.9 numerical aperture to focus the laser onto the perovskite film with a spot of about 1 micron in size. PL was collected in backscattering mode. After passing through a Raman filter set (to eliminate the laser component), the PL was redirected through a multimode optical fiber to a lens-based (300 mm focal length) UHTS300 spectrometer, dispersed with a 600 lines per mm grating, blazed at 500 nm, and collected using a Peltier-cooled front-illuminated charge coupled device (CCD) camera. Due to the large PL efficiency of the perovskite, an extremely low laser power of 0.9 μW and 4 μW for the 633 nm and the 785 nm line, respectively, was employed to collect PL spectra without saturating the CCD camera and avoiding any photo-degradation of the perovskite film. The acquisition time was set to 100 ms per point. The PL images typically consisted of square regions with sizes ranging from 25 × 25 to 50 × 50 μm^2^, analyzed in lateral steps of 1 to 2 μm, thus collecting up to a total of 2500 spectra per image. The analysis of the PL images was performed using the WITec Project FIVE software.

## Conflicts of interest

There are no conflicts to declare.

## Supplementary Material

EE-012-C9EE00884E-s001
