# Supplementary material for: Ferroelectricity-free lead halide perovskites
Source: Energy Environ Sci. 2019 Jun 21;12(8):2537–47. doi: 10.1039/c9ee00884e (PMC8522734; doi:10.1039/c9ee00884e)
Supplement: EE-012-C9EE00884E-s001 [file EE-012-C9EE00884E-s001.pdf]

Electronic Supplementary Material (ESI) for Energy & Environmental Science.
This journal is © The Royal Society of Chemistry 2019

Supplementary Information

Ferroelectricity-free Lead Halide Perovskites

Andrés. Gomez[1*], Qiong. Wang[2*], Alejandro. R. Goñi[1,3], Mariano Campoy-Quiles[1], Antonio. Abate[2,4,5*]

[1] Instituto de Ciencia de Materiales de Barcelona (ICMAB-CSIC), Campus UAB, 08193 Bellaterra, Spain

[2] Helmholtz-Zentrum Berlin für Materialien und Energie, Kekuléstrasse 5, 12489 Berlin, Germany

[3] ICREA, Passeig Lluís Companys 23, 08010 Barcelona, Spain

[4] Institute of Advanced Energy Materials, Fuzhou University, Fuzhou, Fujian 350002, China.

[5] Department of Chemical, Materials and Production Engineering, University of Naples Federioco II, Piazzale Tecchio 80, 80125 Fuorigrotta, Naples, Italy.

*Corresponding authors: AG agomez@icmab.es; QW qiong.wang@helmholtz-berlin.de; AA antonio.abate@helmholtz-berlin.de; antonio.abate@unina.it

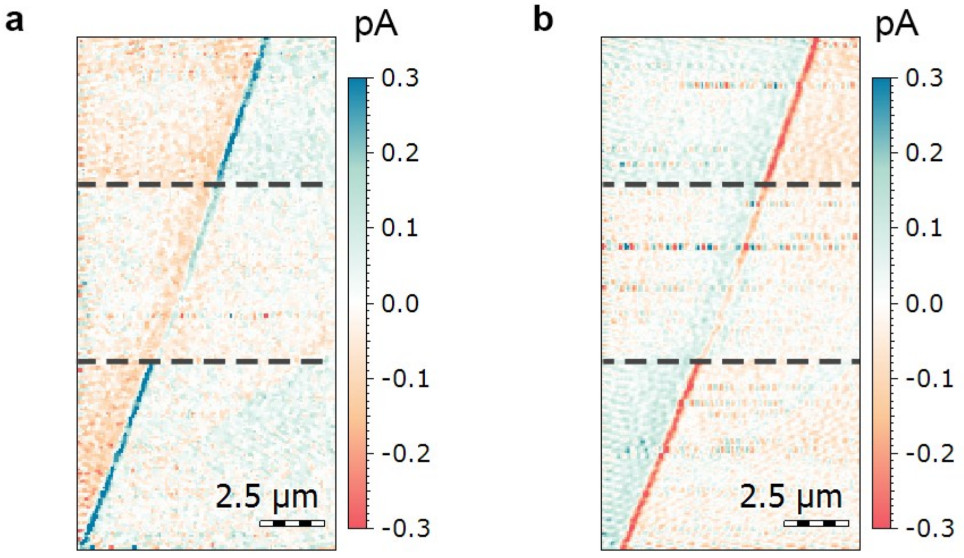

Figure S1. DPFM of PPLN performed in the same conditions as Figure 2 in the main text. a) DPFM-Si and **b)** DPFM-So respectively. We can see that the current is inversed its sign upon crossing different domain configurations. Black dashed horizontal lines represent the changes in applied force.

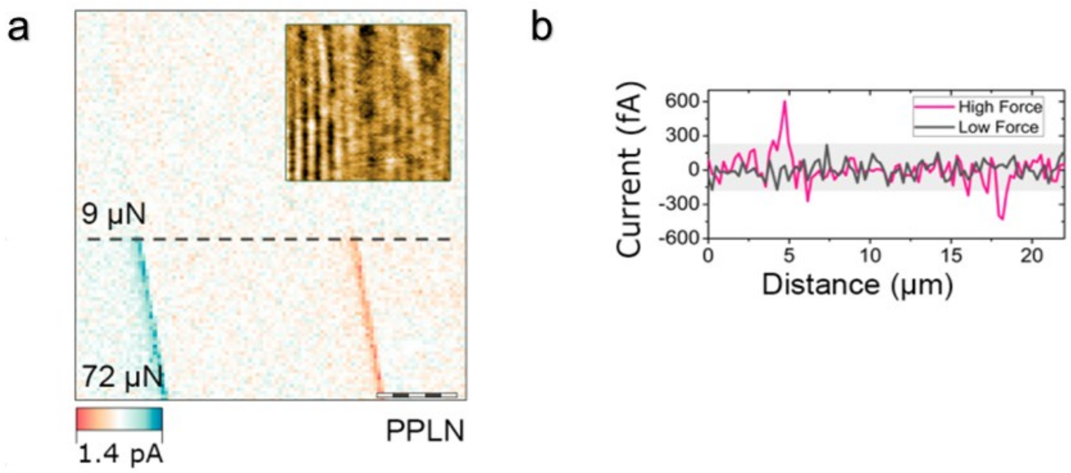

Figure S2. Force dependence of the DPFM signals of PPLN (a) with randomly selected profiles for a high and a low applied force in (b).

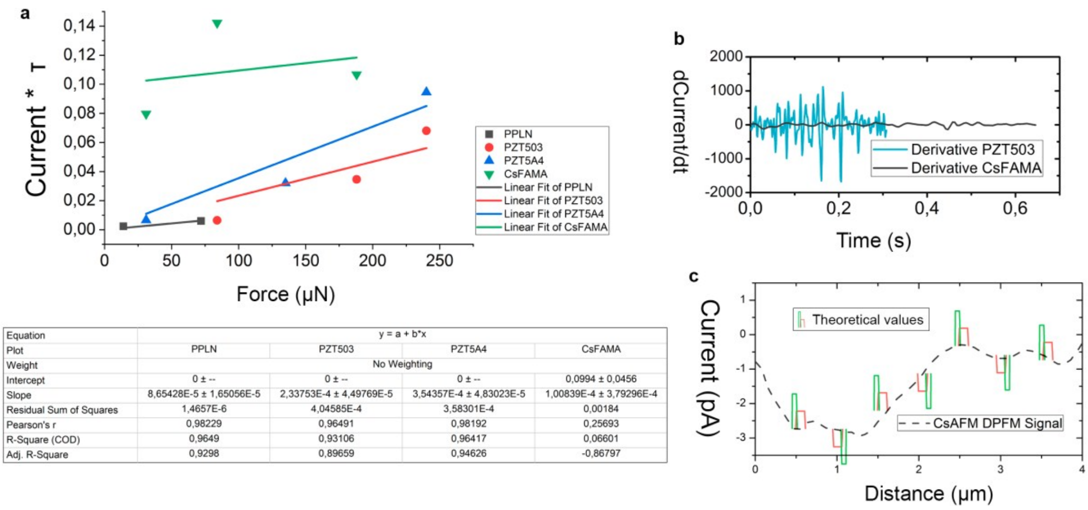

Equation	y = a + b*x			
Plot	PPLN	PZT503	PZT5A4	CsFAMA
Weight	No Weighting			
Intercept	0 ± --	0 ± --	0 ± --	0,0994 ± 0,0456
Slope	8,65428E-5 ± 1,65056E-5	2,33753E-4 ± 4,49769E-5	3,54357E-4 ± 4,83023E-5	1,00839E-4 ± 3,79296E-4
Residual Sum of Squares	1,4657E-6	4,04585E-4	3,58301E-4	0,00184
Pearson's r	0,98229	0,96491	0,98192	0,25693
R-Square (COD)	0,9649	0,93106	0,96417	0,06601
Adj. R-Square	0,9298	0,89659	0,94626	-0,86797

Figure S3. Extracted from the data given in Figure 2 for samples of PPLN, PZT 503/5A4 and CsFAMA perovskite. a) Dependence of (current* τ) on applied force obtained from Figure 2. Linear dependency can be found in known ferroelectric samples, *i.e*. PPLN and PZT503/5A4. However CsFAMA perovskite cannot be fitted linearly. τ corresponds to the pixel time constant of each image. b) Numerical derivative of the current profiles *v.s*. time (s) for PZT503 and CsFAMA perovskite. Since piezogenerated current shows a peak only when the tip crosses different domain structures, the derivative is crisp while in the case of CsFAMA perovskite, the derivative is rather flat. c) Current profiles for CsFAMA perovskite as depicted in Figure 2d in which the theoretical calculated piezogenerated peaks are found. The peaks assume a d_{33} constant of 5 pC/N, an applied force of 200 µN with different tip-sample contact radius of 100 nm for red line and 50 nm for green line.

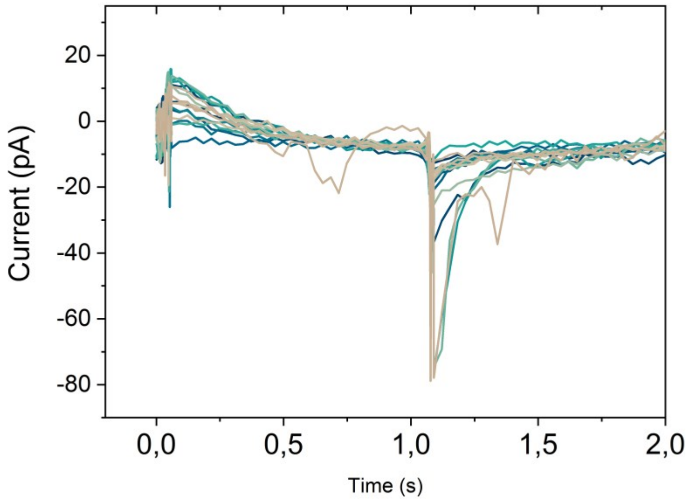

Figure S4. Results of the 16 curves obtained in DPFM spectroscopy experiment of CsFAMA perovskite. Each curve presents a single spot performed in an array of 4x4 points of a 10x10 micrometers area. Despite the highly localized measurement conditions, we can see that there is a clear tendency of the slow decay of the current which cannot be associated to piezoelectric effect.

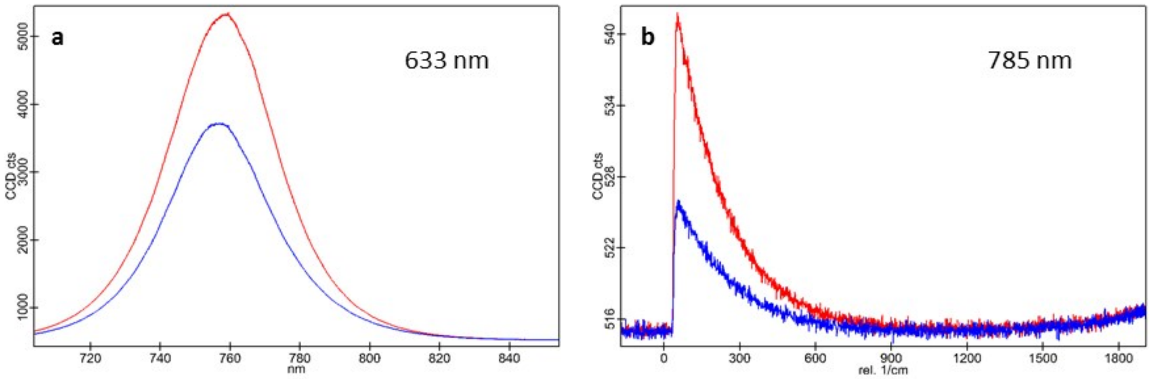

Figure S5. PL spectra of a pre-patterned perovskite film. a) Steady-state PL spectra of CsFAMA perovskite film with an excitation wavelength at 633 nm measured outside (red trace) and inside (blue trace) the pre-scanned area by the AFM tip with a constant force. **b)** Transient PL measurement of CsFAMA perovskite with an excitation wavelength at 785 nm.

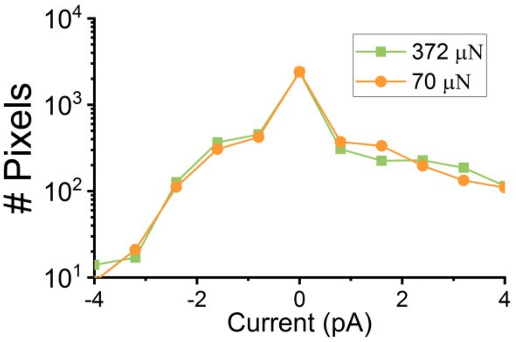

Figure S6. Histograms for $MAPbI_3$ film obtained with images of Figure 4b at high (green line with square symbols) and low (organ line with circle symbols) applied forces. Despite one applied force is around 5 times higher than the other, the current signals at the two applied force almost overlapped with each other.

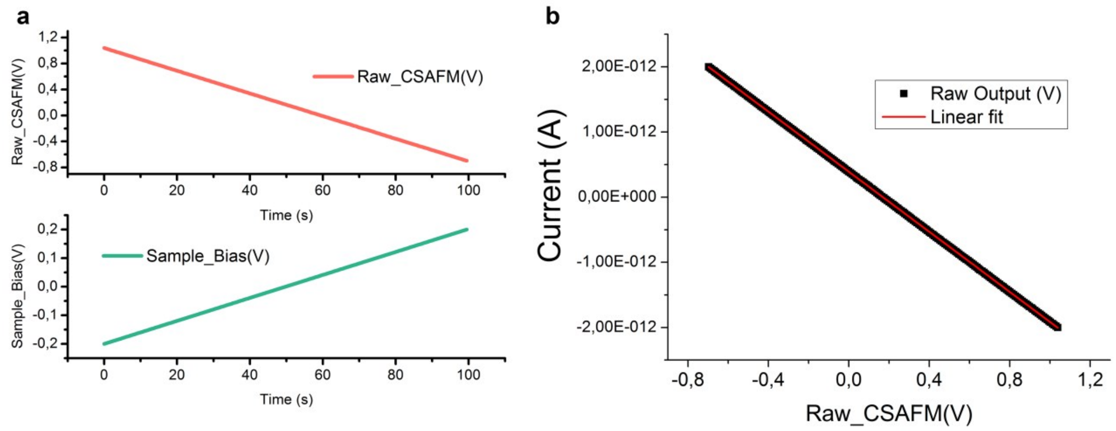

Figure S7. Electrical calibration of the amplifier used. **a)**, plotting of the output of our amplifier (Raw_CSAFM(V)) vs Time while applying the sample bias showed. **b)** correlation of the theoretical current circulating over the system (there is a known test resistor of 100 G Ohm) vs the Output signal of the amplifier. A linear fit is employed to find the exact calibration value of the amplifier (-2.30E-12 +- 2.5E-16).

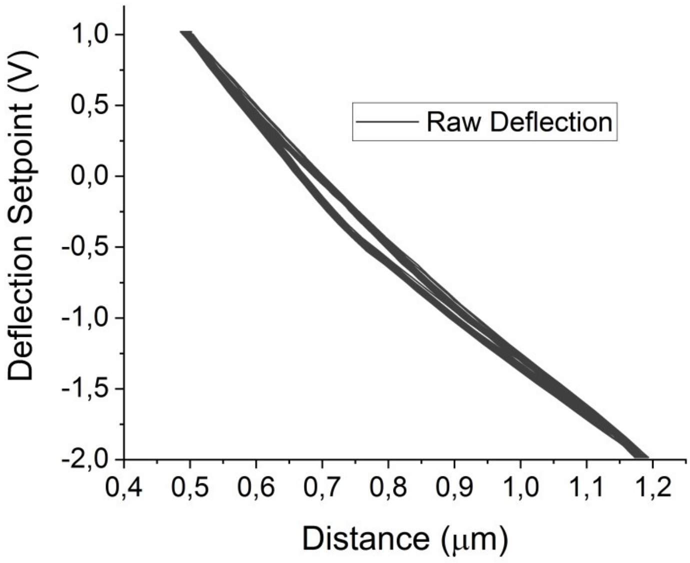

Figure S8. Force-vs-Distance curves obtained for different points in the sample. from this data we are able to obtain the deflection sensitivity, to calibrate the setpoint value in distance units. With the spring constant of the cantilever, we are able to obtain the exact force applied.

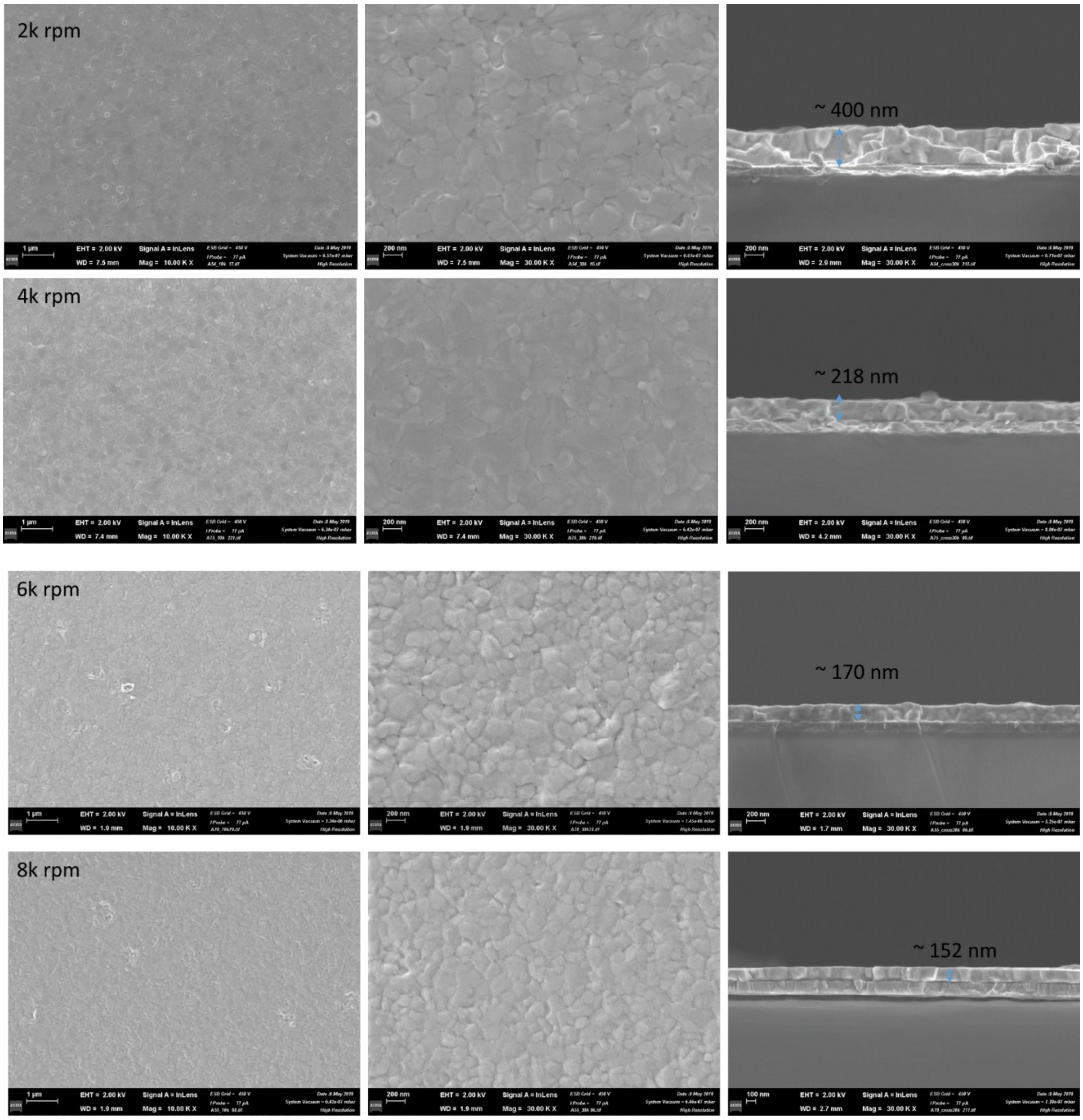

Figure S9. Surface morphology and cross-sectional images of $MAPbI_3$ perovskite films deposited at different spin coating speeds, *i.e.* 2k rpm, 4k rpm (control), 6k rpm, and 8k rpm.

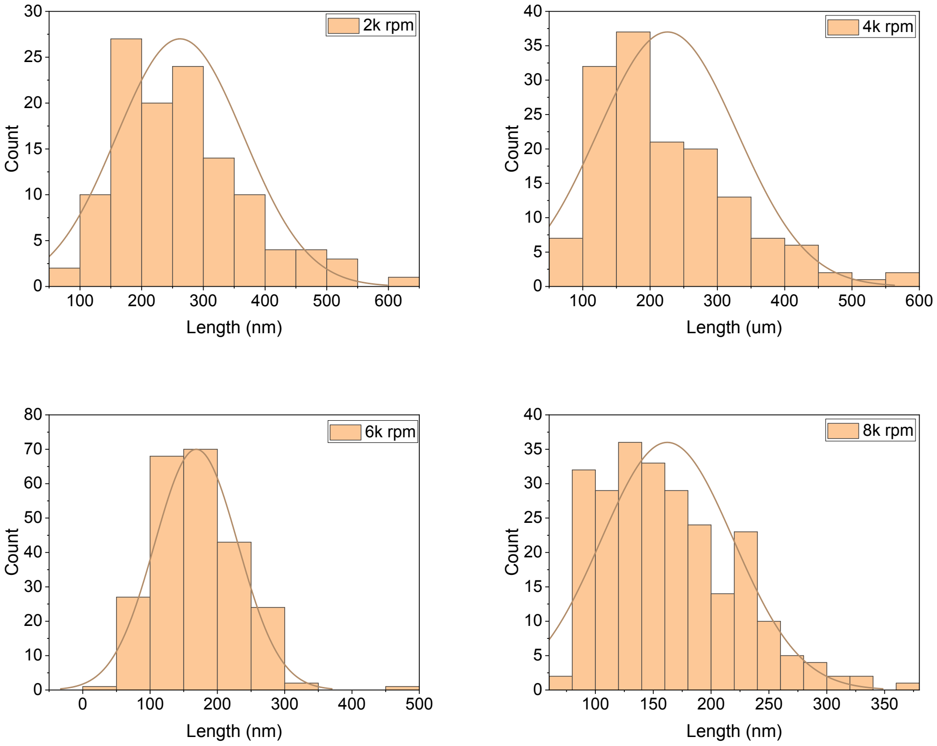

Figure S10. Grain size distribution of $MAPbI_3$ perovskite films given in **Figure S9**.

a)

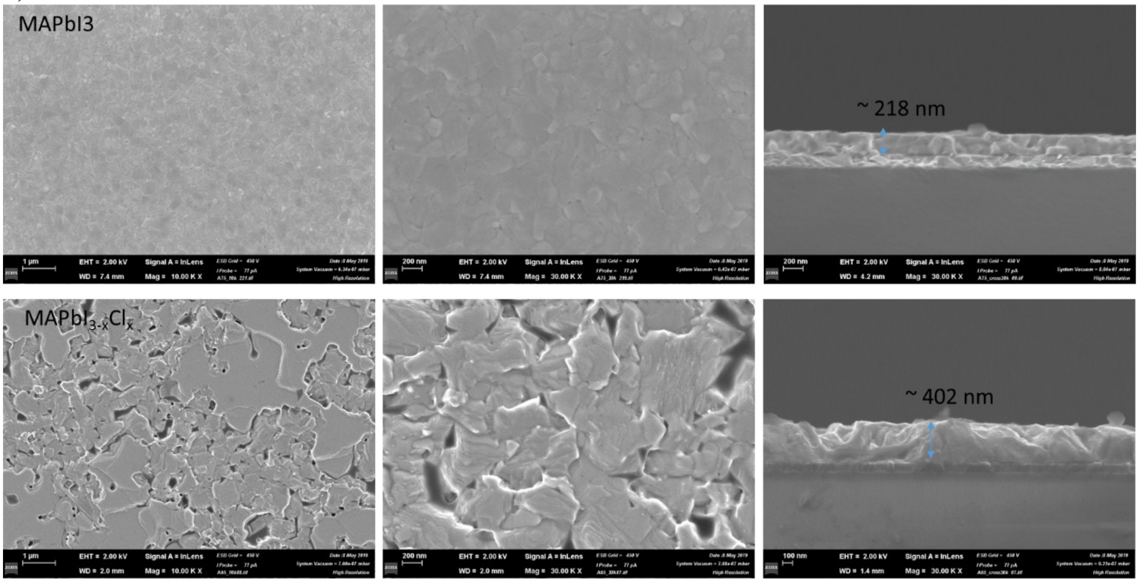

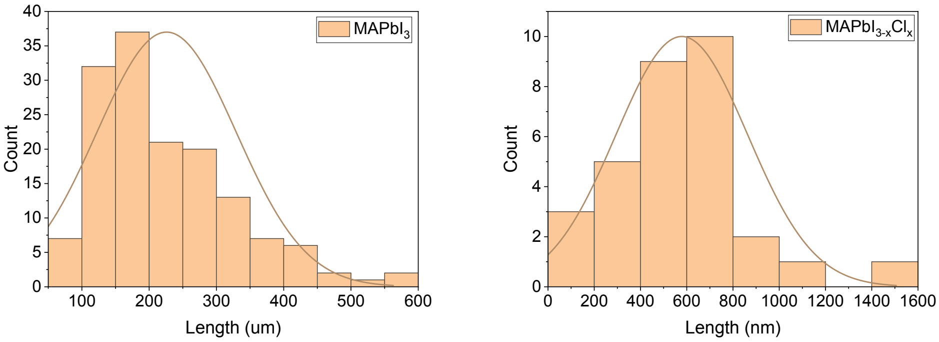

b)

Figure S11. a) Surface morphology and cross-sectional images of $MAPbI_3$ perovskite films deposited from precursors with and without chlorine. b) Grain size distribution of $MAPbI_3$ perovskite films given in a).

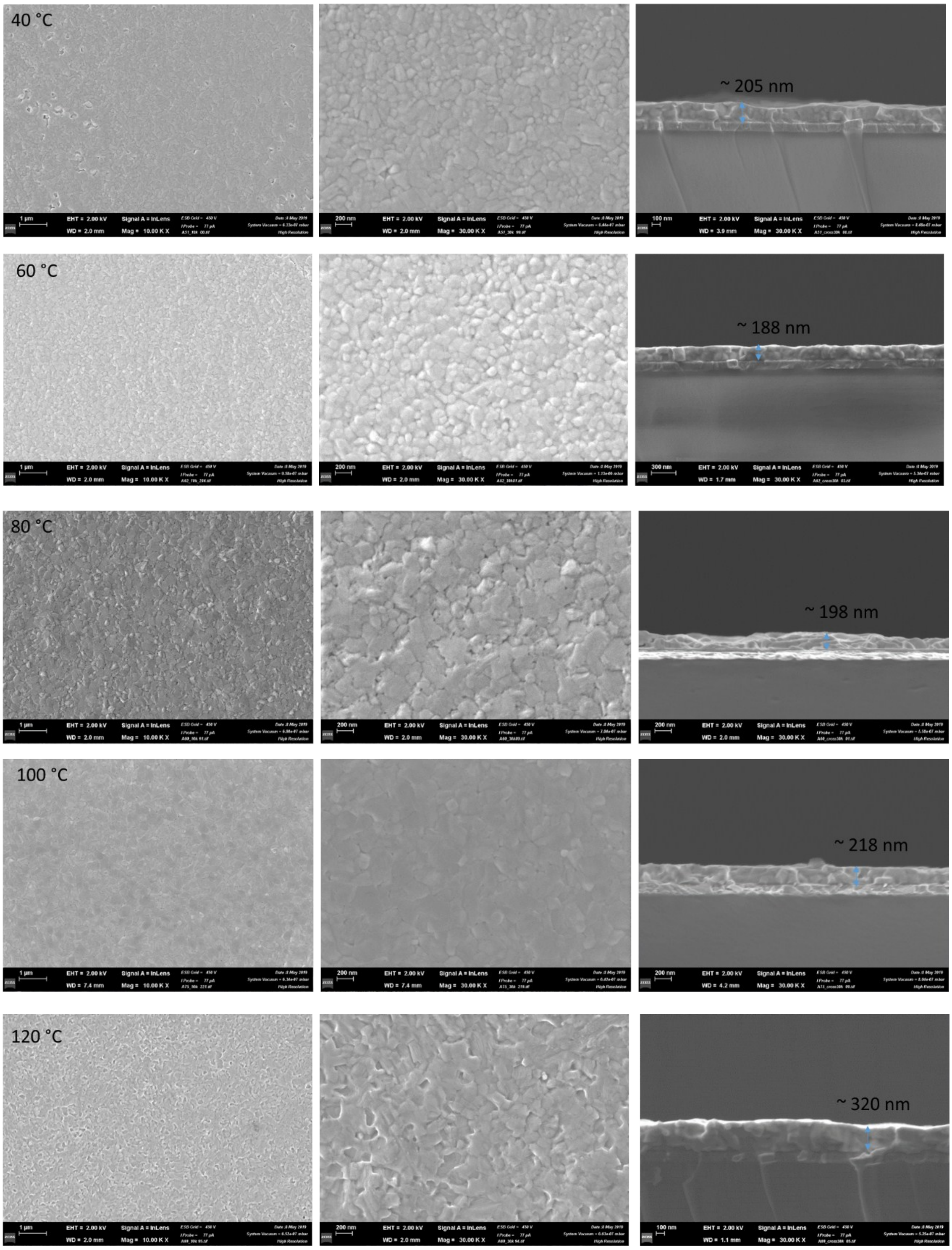

Figure S12. Surface morphology and cross-sectional images of $MAPbI_3$ perovskite films annealed at different temperatures, *i.e.* 40 °C, 60 °C, 80 °C, 100 °C, and 120 °C.

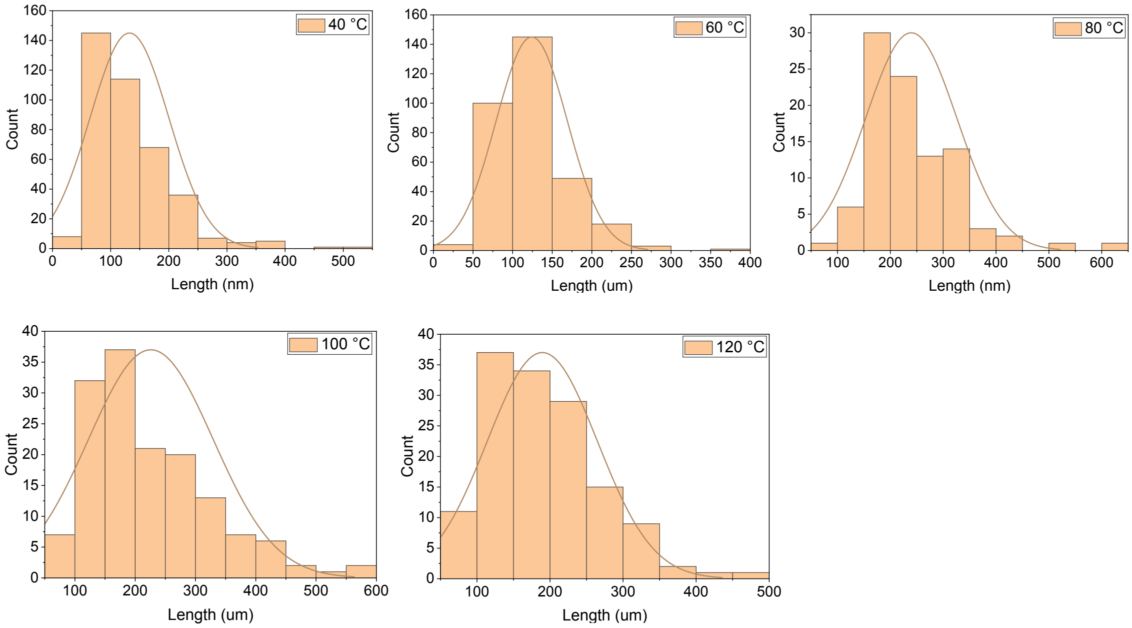

Figure S13. Grain size distribution of $MAPbI_3$ perovskite films given in **Figure S12**.

a)

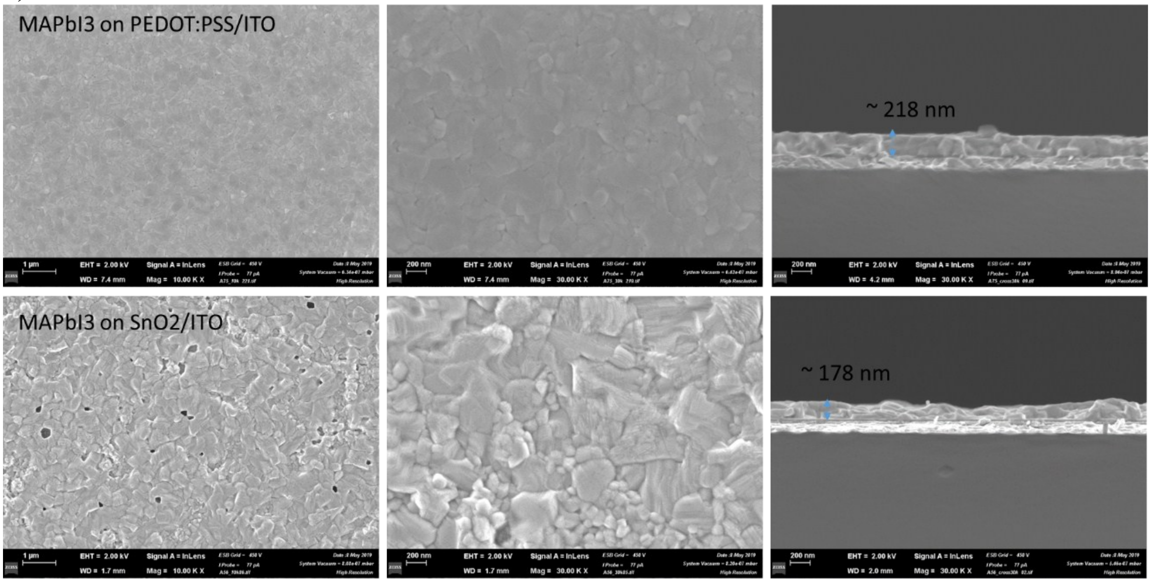

b)

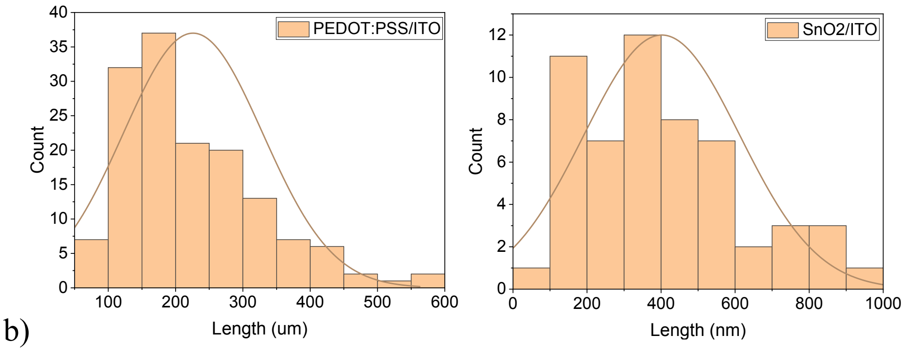

Figure S14. a) Surface morphology and cross-sectional images of $MAPbI_3$ perovskite films deposited on different substrates, *i.e.* PEDOT:PSS covered ITO (top) and SnO_2 covered ITO (bottom). b) Grain size distribution of $MAPbI_3$ perovskite films given in a).

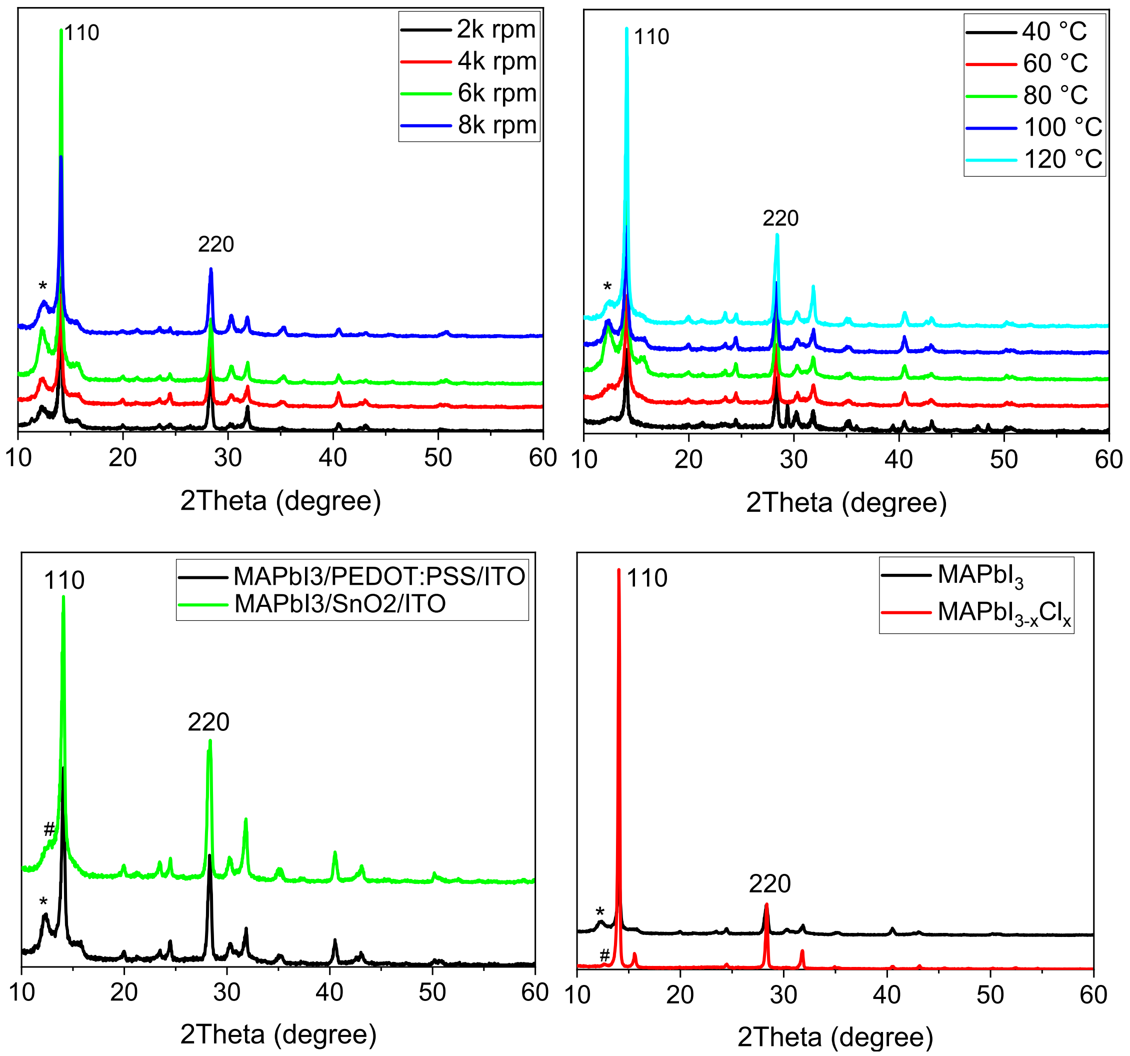

Figure S15. X-ray diffraction (XRD) pattern of $MAPbI_3$ perovskite films of different spin-coating speeds, annealing temperatures, substrates, and precursors with and without chlorine.
